# Potential identity of multi-potential cancer stem-like subpopulation after radiation of cultured brain glioma

**DOI:** 10.1186/1471-2202-9-15

**Published:** 2008-01-30

**Authors:** Mi K Kang, Beong I Hur, Mi H Ko, Cheul H Kim, Seung H Cha, Soo K Kang

**Affiliations:** 1 Department of Physiology, College of Medicine, Pusan National University, Busan, Korea; 2 Department of Neurosurgery, College of Medicine, Pusan National University, Busan, Korea; 3 Department of Anesthesiology, College of Medicine, Pusan National University, Busan, Korea

## Abstract

**Background:**

Glioblastoma multiforme (GBM) is the most frequently encountered brain cancer. Although the existence of cancer stem cells in GBM has been previously established, there is little evidence to explain the difference between cancer stem cells and radio-resistant cells in GBM. In an effort to increase our understanding of whether cellular radio-resistance is a characteristic associated with cancer stem cells, we developed a dissociated cell system of subpopulations derived from GBM, and demonstrated radiotherapy resistance therein.

**Results:**

The radio-resistant cancer cell subpopulations of GBM abundantly express CD133, CD117, CD71, and CD45 surface markers, and these radio-resistant cancer cell subpopulations have the capacity for extensive proliferation, self-renewal, and pluripotency. These radio-resistant cancer subpopulations have been shown to initiate tumorigenesis when transplanted into SCID mouse brains. Moreover, these tumors evidenced highly peculiar nest-like shapes harboring both vascular and cancerous tissue structures, which expressed the blood vessel specific marker, the von Willebrand factor. Accordingly, subpopulations of radio-resistant cells in GBM have been shown to be very similar to hematopoietic stem cells (HSCs) in the circulating blood. This similarity may contribute to increased tumor growth and GBM recurrence.

**Conclusion:**

The results of the present study provide further evidence for radio resistant subpopulations of cancer stem cells in GBM. Also, our results will assist in the identification and characterization of cancer stem cell populations in glioma, and will help to improve the therapeutic outcomes of GBM.

## Background

In the past few years, it has been reported that cancers tend to harbor small cell populations with the capability to sustain tumor formation and growth in tumor cells. These cells are referred to as cancer stem cells (CSCs) [1,2]. CSCs have been identified in leukemia [3], multiple myeloma [4] and breast cancer [5,6]. Recently, several studies have demonstrated the existence of CSCs in human brain tumors [7-9]. These CSCs shared many properties, including self-renewal and multi-potency, with normal stem cells [10,11], and expressed a wide variety of transporters involved in drug efflux [12-14]. Moreover, CSCs can initiate tumors when transplanted into immune-deficient mice [10,11].

As compared with other brain tumors, glioblastoma multiforme (GBM) is a relatively aggressive variant in humans [15]. A combination of surgery, radiotherapy, and chemotherapy comprises the standard treatment in such cases [16]. Despite constant efforts to develop prevention protocols for GBM, GBM is intrinsically resistant to conventional therapies, including radiotherapy and chemotherapy [12]. Singh *et al*. [11,17] previously isolated a CD133^+ ^cell population from glioma that evidenced properties of CSCs *in vitro*, and initiated tumors *in vivo*. In a recent work conducted by Bao *et al*. [18], it was shown that CD133^+ ^glioma stem cells contributed to glioma radioresistance via the preferential activation of DNA damage checkpoint response and an increase in DNA repair capacity as compared to CD133^- ^tumor cells. Therefore, the identification of the cell types involved in resistance phenomena is critical from both a scientific and therapeutic standpoint in cases of GBM. Although the existence of cancer stem cells in GBM has been firmly established, there is currently little evidence to explain the difference between cancer stem cells and radio-resistant cells in GBM. In this study, we have attempted to determine whether cellular radio-resistance is associated with cancer stem cells in GBM. For a better understanding of GBM, we developed a dissociated cell system to facilitate the study of cancer stem cell subpopulations derived from GBM, which evidenced resistance to radiotherapy. We observed that radio-resistant cancer stem cell subpopulations of GBM expressed CD133, CD117, CD71, and CD45 cell surface markers, and also evidenced downregulated neural marker expression. Also, these radio-resistant stem cell subpopulations have been shown to initiate tumorigenesis when stereotaxically transplanted into SCID mouse brains. These findings provide further evidence for the existence of radio-resistant subpopulations in GBM CSCs. Our results will also help us to understand the properties of radio-resistant subpopulation and help to improve the therapeutic outcomes of GBM.

## Results

### Brain cancer cells have the resistant ability toward radiation

**Figure 1:**
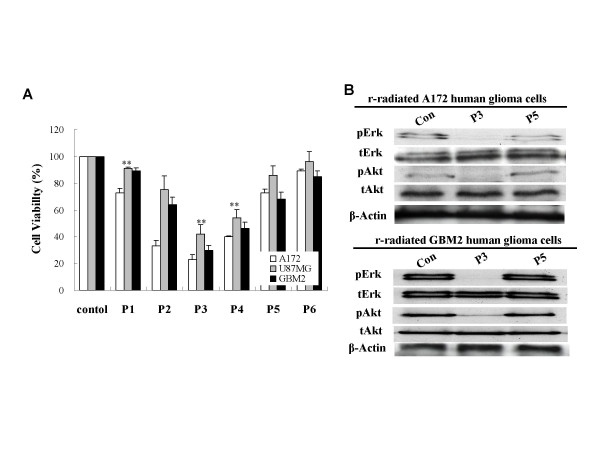
**Cell proliferation of human glioblastoma cells by gamma-irradiation**. The cell number was measured at various culture passages after gamma-irradiation through tryphan blue exclusion method. (A) Relative cell viability of A172, GBM2, and U87MG cells at various culture passages after gamma-irradiation. (B) Western blot analysis of total cell extractions isolated from human glioblastoma cells, A172 and GBM2, at various culture passages after gamma-irradiation. Results of triplicate samples were expressed as mean ± SD. **p < 0.01 vs control.

**Figure 2:**
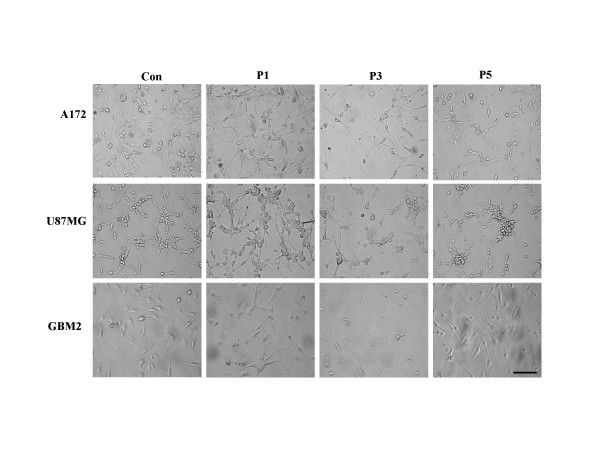
**Morphological change of human glioblastoma cells by gamma-irradiation**. Phase microphotographs of cultured radio-resistant tumor cells of A172, GBM2, and U87MG cells (× 200 magnification) at various culture passages after gamma-radiation. Scale bars, 50 μm.

**Figure 3:**
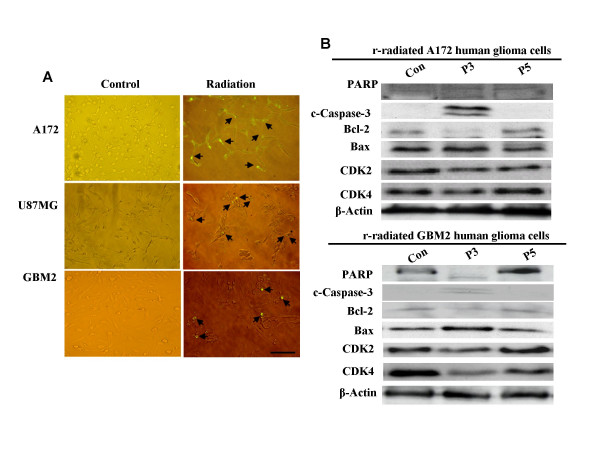
**Apoptotic cell death of human glioblastoma cells as the result of gamma-irradiation**. (A) TUNEL assays of A172, GBM2, and U87MG cells following gamma-irradiation (× 200 magnification). Arrows indicate the apoptotic deaths of the tumor cells. (B) Western blot analysis of total cell extractions isolated from A172 and GBM2 at various culture passages after gamma-irradiation. Scale bars, 50 μm.

Radiation exposure has been shown previously to reduce the survival rates of various glioblastoma cell lines – including A172, U87MG, and GBM2. As is shown in Fig. 1, the majority of cells perished during the culture period, leaving only a few surviving cells. After 3 passages of subsequent applications, the survival rates of A172, GBM2, and U87MG were 23, 42, and 30%, respectively (Fig. 1A). The cell line most sensitive to radiation was A172, followed by GBM2, whereas U87MG was the cancer cell line most resistant to radiation. However, the surviving cells proliferated with additional culture time. It was determined that small cell populations of the GBM cell lines were resistant to radiation, and that these resistant cells could actively proliferate. As is shown Fig. 2, the three GBM cell lines evidenced spindle-shaped cells after radiation, and progressively deteriorated and perished during the culture periods. After radiation, only small cell populations evidencing a round morphology remained by the end of the third culture period (Fig. 2). In addition, these small cells proliferated and repopulated as the result of subsequent cultures (Fig. 1A). Similarly in morphological changes occurring in gamma-irradiated glioblastoma cells, Erk1/2 and Akt phosphorylation was reduced in A172, GBM2, and U87MG cells in the third culture period post-radiation, but recovered in subsequent culture passages (Fig. 1B). These results showed that some of the cells evidencing resistance to radiation in the GBM cell lines were capable of proliferating via the activation of Erk1/2 and Akt. When the three types of GBM cells were treated with gamma irradiation, apoptotic events were observed via the TUNEL assay process (Fig. 3A). In addition, the cleaved forms of caspase-3 and PARP were increased as the result of radiation. Subsequently, Bax protein expression was increased and Bcl-2 protein expression was attenuated by gamma-irradiation at the same culture passage. However, these protein expressions were recovered over a prolonged culture period (Fig. 3B). It was suggested that the radio-sensitive population of cancer cells inhibited the growth of cells via the induction of apoptosis. On the other hand, a few radio-resistant GBM cells survived and proliferated after radiation, via an ability to escape apoptosis. In an effort to further determine whether radio-resistant GBM cells are associated with cancer stem cells, A172 and GBM2 cells were selected for subsequent experiments, because these two types of cells were more sensitive than U87MG.

### Radio-resistant populations of brain cancer cells contain a stem cell-like subpopulation

**Figure 4:**
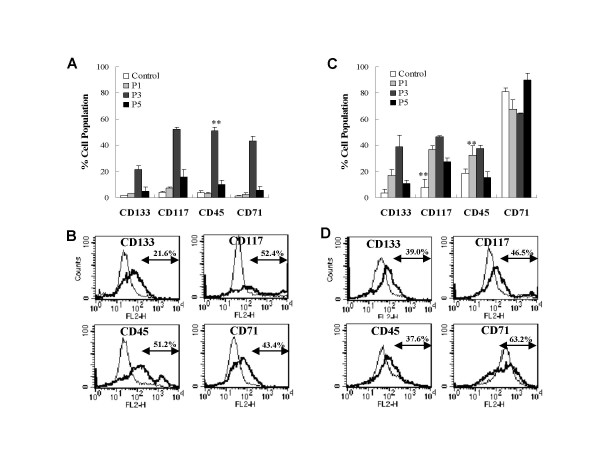
**Surface markers expressions of radio-resistant cells derived from human glioblastoma cells**. The cultured radio-resistant cells (1 × 10^6^) were stained with CD133, CD117, CD45, and CD71 coupled to fluorescein isothiocyanate (FITC) or PE (phycoerythrin) at various culture passages after gamma-irradiation, and then analyzed via flow cytometry. (A) Each cell subpopulation rate of A172 cells after gamma-irradiation. (B) Representative flow cytometry histogram of radio-resistant A172 subpopulations for CD133, CD117, CD45 and CD71 at three culture passages after gamma-irradiation. (C) Each cell subpopulation rate of GBM2 cells after gamma-radiation. (D) Representative flow cytometry histogram of radio-resistant GBM2 subpopulations for CD133, CD117, CD45 and CD71 at three culture passages after gamma-radiation. The results of triplicate samples were expressed as the means ± SD. **p < 0.01 vs control.

Radio-resistant cancer cells of the A172 and GBM2 cell lines were stained with fluorescence-conjugated primary antibodies against surface markers for stem cells, CD133, CD117, CD45, and CD71 according to the post-radiation culture passage, and analyzed via flow cytometry. After radiation, a variety of stem cell surface markers were increased by culture passage, and their maximum rates were achieved at the third culture passage (Fig. 4). As is shown in Fig. 4B, radio-resistant A172 and GBM2 cancer cells harbor 21.6 and 39.0% of CD133+, 52.4 and 46.5% of CD117+, 51.2 and 37.6% of CD45+, 43.4 and 36.2% of CD71+ cells, respectively. However, stains of the surface markers on radio-resistant A172 and GBM2 cells were decreased to control levels after further subcultures. It was determined that a variety of stem cell-like subpopulation cell types may exist in the population of radio-resistant A172 and GBM2 cancer cells, and these may be capable of proliferating on the stem-cell negative population layer.

**Figure 5:**
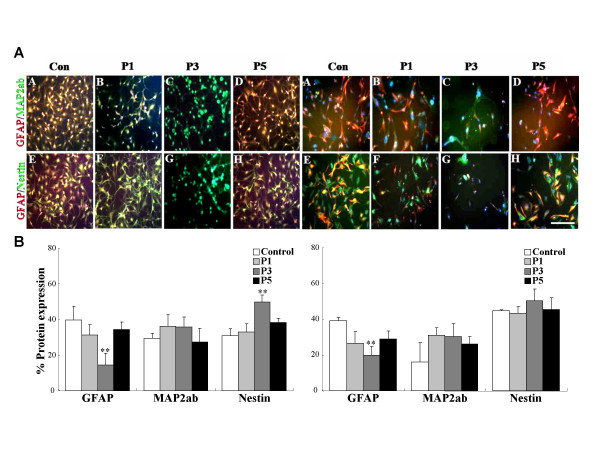
**Immunocytotochemical characteristics of radio-resistant cells derived from human glioblastoma cells**. Cells were labeled with antibodies to Nestin (green) or MAP2ab (green) to recognized neurons or GFAP (red) to recognized glia at various culture passages after gamma-irradiation. (A) Fluorescence microscope images (× 200 magnification) and relative protein expression of radio-resistant A172 cells over the culture passages. (B) Fluorescence microscope images (× 200 magnification) and relative protein expression of radio-resistant GBM2 cells over the culture passages. The results of triplicate samples were expressed as the means ± SD. **p < 0.01 vs control. Scale bars, 50 μm.

To further evaluate the protein expression of radio-resistant subpopulations in the cancer cell population, we conducted double immunostaining analysis according to the passages. The A172 and GBM2 cells were shown to express markers for the NSC marker Nestin (31.0 and 44.8%), the neuronal marker MAP2ab (29.3 and 16.2%), and the astrocyte marker GFAP (39.7 and 39.0%) prior to radiation (Fig. 5). However, the population of GFAP+ cells was decreased significantly after radiation and the amount of Nestin+ cells increased progressively up to the fifth culture passage. These results showed that Nestin+ cells of A172 and GBM2 were more resistant then GFAP+ cells and radio-resistant Nestin+ cells were capable of actively proliferating.

### Radio-resistant stem cell-like subpopulations of brain cancer cells have the ability to multipotency

**Figure 6:**
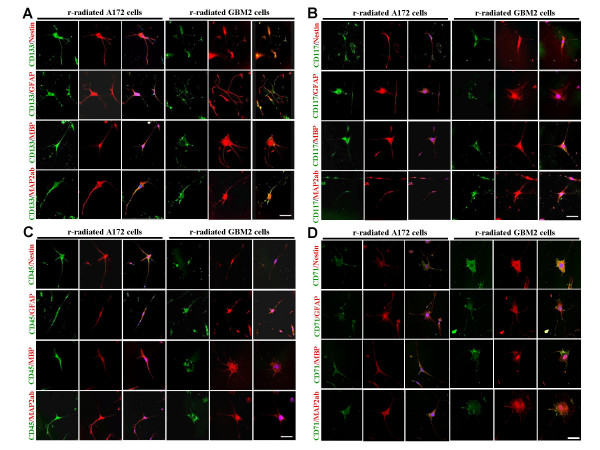
***In vitro *differentiation and cell surface marker coexpression of radio-resistant cells derived from human glioblastoma cells**. Cells were labeled with antibodies for neuronal (Tuj and MAP2ab; red), glial (GFAP; red), and neural stem cells (Nestin; red), and cell surface epitopes (CD133, CD117, and CD45; green), and were then observed via laser scanning confocal microscopy. (A) Laser scanning images of differentiation and CD133 cell surface marker coexpression of radio-resistant human glioblastoma A172 and GBM2 cells. (B) Laser scanning images of differentiation and CD117 cell surface marker coexpression of radio-resistant human glioblastoma A172 and GBM2 cells. (C) Laser scanning images of differentiation and CD133 cell surface marker coexpression of radioresistant human glioblastoma A172 and GBM2 cells. (D) Laser scanning images of differentiation and CD71 cell surface marker coexpression of radioresistant human glioblastoma A172 and GBM2 cells. Images represent × 400 magnification. Scale bars, 25 μm.

The multipotency of individual radio-resistant stem cells-like subpopulations within the A172 and GBM2 cancer cells was directly evaluated via analysis of their neural differentiation potentials (Fig. 6). Not only differentiated radio-resistant stem cell-like subpopulations of A172 cancer cells evidenced immunoreactivity for hematopoietic stem cell surface markers (including CD133, CD117, CD71, and CD45) and nestin, but they also were able to differentiate into GFAP-positive astrocyte-like cells, MBP-positive myelin-like cells and MAP2ab-positive neuron-like cells (Fig. 6). Conversely, in GBM2-derived radio-resistant stem cell-like populations, CD133, CD117, CD71, and CD45 cell surface epitopes coexhibited immunoreactivity for Nestin-positive cells, but lacked immunoreactivity for other differentiated neural cell markers, including GFAP, MBP, and MAP2ab. These findings showed that the stem cell-like population derived from radio-resistant brain cancer cells evidences multipotency and proliferation capacity, and those capacities were definitely different from control cancer cells.

### The in vivo tumorigenic potential of radio-resistant cancer stem cell populations derived from brain cancer cells

**Figure 7:**
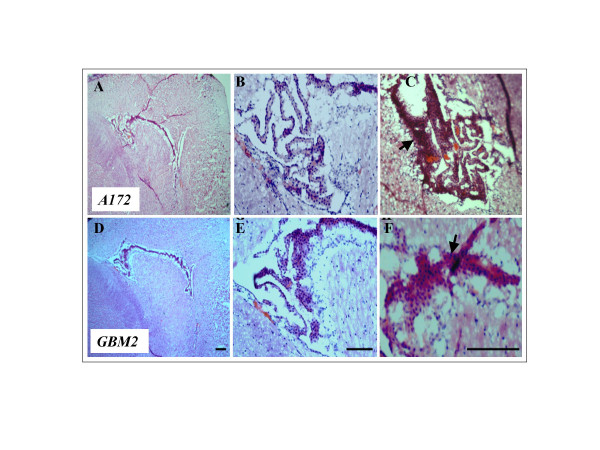
***In vivo *tumorigeneisis of radio-resistant cells derived from human glioblastoma cells by transplanted subpopulation in SCID mouse**. Mice were transplanted with radio-resistant A172 cells (A-C) or GBM2 cells (D-F) into the right striatum via stereotaxic injection, and sacrificed at 4 weeks post-transplantation. Frozen sections of brain tumors were stained with hematoxylin and eosin. (A and F) Peculiar tumor mass-like structure (arrow) and blood vascular formation derived from radio-resistant A172 cells (A, × 40 magnification; B, × 100 magnification) and radio-resistant GBM2 cells (D, × 40 magnification; E, × 100 magnification). (C and F) Solid tumor formation derived from radio-resistant A172 cells (C, × 100 magnification) and radio-resistant GBM2 cells (F, × 200 magnification). Scale bars, 100 μm.

In an effort to determine whether radio-resistant cancer stem cell-like subpopulations of A172 and GBM2 cells were capable of *in vivo *tumor initiation, we transplanted these cells into SCID mouse brain tissue. Radio-resistant cancer stem cell-like subpopulations derived from GBM cancer cell lines were shown to initiate tumor formation below the cell injection site after 4 weeks (Fig. 7). As was determined by hematoxylin and eosin staining, radio-resistant cancer stem cell-like populations generated highly peculiar nest-like tumors harboring both vascular structure (Fig. 7B and 7E) and cancerous tissue structure (Fig. 7C and 7F). The presence of both cell types in the regenerated lesions indicates that lesions derived from the brain cancer stem cells of radio-resistant glioblastoma cells possess a profound capacity for multi-lineage differentiation and the generation of blood vessels and tumor masses in the brain.

**Figure 8:**
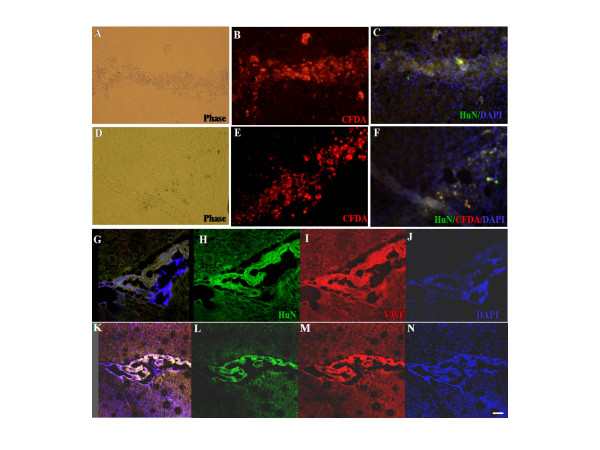
**Immunocytochemical characteristics of brain tumor mass from SCID mouse transplanted with radio-resistant cancer stem cells derived from human gliobalstoma cells**. Frozen sections of brain tumors were labeled with antibodies to human-nuclei to recognize engrafted human gliobalstoma cells (Red) or von Willebrand factor to recognize blood vessels (Green). Fluorescence microscope images of solid tumors from brain tissue transplanted with radio-resistant A172 (C-F) and GBM2 (G-J) cells. (A-B) Demonstrates a human-nuclei (red) and von Willebrand factor of solid tumors as the result of transplantation with radio-resistant cells (× 100 magnification). The sections were also labeled with DAPI (blue) to identify nuclei. Laser scaning confocal images of blood vascular tissue from brain samples by transplantation with radio-resistant A172 (C-F) and GBM2 (G-J) cells (× 100 magnification). (C-J) Demonstrates a von Willebrand factor (green), human-nuclei (red) of blood vascular, and TOPRO-3 (blue) derived from radio-resistant A172 (C-F) and GBM2 (G-J) cells (× 100 magnification). G and K are merged Confocal images derived from radio-resistant A172 (C) and GBM2 (G) cells, respectively. Scale bar, 50 μm.

The regenerated tumors evidenced immunoreactivity for typical human-nuclei specific antigens (Fig. 8A–F) and for the blood vessel-specific marker, von Willebrand factor (Fig. 8G–N). Also, immunohistochemical analysis verified that these tumors were derived from engrafted radio-resistant cancer stem cells and displayed a profound proliferating activity, as evidenced by DAPI. Therefore, the presence of regenerated tumors in transplanted SCID mouse brain tissue suggested that the radio-resistant cancer cells possess a profound tumorigenic capacity.

## Discussion and Conclusion

Glioblastoma multiforme (GBM) represents one of the most frequently occurring brain cancers [15]. Besides surgery, which is an important initial therapeutic measure in cases of malignant glioma, postoperative radiotherapy, including modern treatment techniques, is considered to be the standard treatment in these cases [19-21]. Radiation is the most frequently used in the treatment of GBM. Today, the data from Walker *et al *[22], which indicated a doubling of the median survival time in GBM from 4–5 months to 9–12 months with the application of postoperative radiotherapy, remain valid. However, radiotherapy does not appear to substantially prolong the median survival rates of GBM patients. Moreover, GBM harbored subpopulations of cells with intrinsic resistance to radiation, and these may be capable of repopulating tumors after treatment [15]. In this study, we determined that only a few A172, GBM2, and U87MG cells survived after exposure to radiation (Fig. 1). After radiation, round-shaped radio-resistant cells proved able to proliferate via the activation of Erk1/2 and Akt (Fig. 1 and 2), whereas the majority of spindle-shaped radio-sensitive cells continued to perish via apoptotic induction (Fig. 3). These findings showed that the growth of the radio-sensitive population of GBM cells was inhibited via apoptotic induction. However, a few radio-resistant GBM cells survived this process, and proliferated after radiation via their ability to escape the apoptosis activation of the proliferation signals [23-25]. In a recent study, it was determined that glioma stem cells contributed to glioma radioresistance via the preferential activation of the DNA damage checkpoint response [18] Therefore, further additional studies will be required in order to elucidate the radioresistance mechanisms of CSCs in GBM.

Recently, CSCs with a capacity of self-renewal and multi-lineage differentiation have been isolated from tumors of the human central nervous system (CNS) [7,8,25] These cells harbor a small subpopulation and can generate all kinds of mature cancer cell populations in culture [12]. The results of our FACS analyses showed that radio-resistant GBM cells contain a variety of subpopulations, which express CD133, CD117, CD45, and CD71 surface protein (Fig. 4). In addition, double immunostaining showed that Nestin-positive cells were more resistant to radiation then GFAP- and MAP2ab-positive cells (Fig. 5). Furthermore, these individual radio-resistant cells were differentiated into astrocytes, myelin, and neurons (Fig. 6). These results showed that radio-resistant GBM subpopulations are cancer stem cells with proliferation and pluripotency abilities.

Numerous investigators have previously utilized antibodies in order to cluster CD markers, thus characterizing and isolating stem cells from a variety of tissues and cell types, based on the profiles of their cell surface antigens. CD133, CD117, and CD45 have been used to identify human hematopoietic stem cells (HSCs) [25]. CD133, in particular, has been used as a marker of brain cancer stem cells, because the subpopulation of CD133+ cells from human brain tumors has been shown to harbor stem cell properties for proliferation, self-renewal, and differentiation *in vivo*, and can also initiate tumors *in vitro *[11,17]. In our study, we determined that radio-resistant cancer stem cell-like subpopulations derived from GBM were primarily Nestin-positive cells which abundantly expressed CD133, CD117, CD45, and CD71 cell surface markers (Fig. 4 and 5). In addition, these radio-resistant cancer stem cell-like subpopulations can initiate tumor and vascular generation when transplanted into SCID mouse brains using the stereotaxic system (Fig. 7 and 8). These findings indicated that CD117, CD45, and CD71, as well as CD133, can be novel markers for human GBM.

Because CD133-positive populations of brain cancer cells were also detected in normal neural stem cells, the cell of origin for a given brain tumor may be normal stem cells [15,26]. Multiple reports have indicated that rodent and human bone marrow (BM) and BM-HSCs can acquire neural cell fates both *in vitro *and *in vivo *[27-30]. Cancer stem cells are thought to be derived from their normal stem cell counterparts [17]. However, our immunohistochemical results showed that radio-resistant stem cell-like subpopulations from GBM evidenced a lack of immunoreactivity against Nestin for neural stem cell types, GFAP for astrocytic cells, and MAP2ab for neurons, according to the culture periods employed (Fig. 6). Furthemore, regenerated tumors in SCID mouse brain tissue were shown to contain vascular structures in addition to cancerous tissue structures (Fig. 7), and also exhibited immunoreactivity for the blood vessel-specific marker, von Willebrand factor (Fig. 8). Similar findings were reported by Massengale *et al*. [31], who determined that hematopoietic stem cells (HSCs) that enter the adult mouse brain become microglia or evidence other hematopoietic cell fates, and their progeny retain lineage fidelity within the CNS. In a recent work conducted by Salmaggi *et al*. [32], it was shown that GBM-derived CSCs contributed to glioma angiogenesis via secretions of vascular endothelial growth factor (VEGF) and CXCL12. Therefore, in our present study, it was shown that radio-resistant stem-like GBM cells are quite similar to HSCs in circulating blood, and they may exacerbate tumor growth and the recurrence of GBM in the brain.

In this study, we developed a dissociated cell system to facilitate the identification and characterization of cancer stem cell subpopulations of GBM evidencing radiotherapy resistance. We also created a model that can be efficiently used for the study of the tumorigenesis properties of cancer stem cells. The cellular heterogeneity of GBM indicated that radio-resistant subpopulations of GBM stem cells expressed CD133, CD117, CD71, and CD45 cell surface markers, and that these radio-resistant cancer stem cell subpopulations are capable of regenerating tumors *in vivo*. Finally, we are able to conclude that radio-resistant cancer stem cells are likely to be the most critical target in treatments for GBM, and a thorough understanding of its biology might allow it to be selectively targeted, thereby greatly improving therapeutic outcomes. Additionally, the notion that the radio-resistant subpopulation of GBM cells is derived from cancer stem cells might result in the development of new strategies for the development of anticancer therapies.

## Methods

### Animals

Female NOD-SCID mice (5-week-old females) were purchased from the Jackson Laboratory. The animals were kept under day/night rhythm and were fed as labium throughout the experimental period. The Pusan National University Committee on Animal Research approved the experimental procedures.

### Cell line and culture conditions

Human glioblastoma multiforme cell lines, A172 and U87MG were obtained from American Type Culture Collection (ATCC, Manassas, USA). The glioblastoma multiforme-derived cell line GBM2 was established in the author's laboratory from a surgical specimen of a 62-old-man. The cells were maintained in Dubelcco's modified Egale's medium (Gibco, USA) supplemented with 10% FBS (Gibco, USA) and 100 units/ml penicillin-streptomycin (Gibco, USA). In all experiments, cells were maintained in 100-mm culture dishes at 37°C i na humidified 5%/CO_2 _95% air atmosphere. GBM2, established cell line from tumor specimen of patients were used at passage 30–35 for the experiments. All of our experiments were performed on cultures that were at 70% confluence, when cells still in log phase growth.

### Radiation exposure

Radiation was performed with a Leksell Gamma Knife (Model 23004 B-2, Elekta Instruments, Sweden) using a ^60^Co gamma source, a dose intensity of 3.14 Gy/min and 400 mm distance between source and target up to a single dose of 30 Gy. We used three kinds of cultured glioblastoma cells, GBM2, A172, and U87MG cells and volume was 4 mm and maximal irradiation dose was 30 Gy per sample. After irradiation, the cells were seeded at 1.0 × 10^6 ^cells/10 cm culture dish in 10 ml DMEM supplemented with 10% FBS. The irradiation culture dishes were usually split twice per week and replaced with the medium containing 10% FBS during subculture. At different time periods (culture passage) after irradiation, the cell number was measured using tryphan blue exclusion method.

### TUNEL (TdT-mediated dUTP-biotin nick end-labeling) assay

The effect of gamma irradiation on the induction of apoptosis was detected with the TdT in situ Apoptosis Detection Kit (Roche, USA), according to the manufacturer's specifications. After irradiation, three types of glioblastoma cells (1 × 10^5 ^cells) were cultured in chamber slide with DMEM containing 10% FBS for 24 h. Then, the cells were fixed with 4% paraformaldehyde. After washing with PBS, the cells were incubated in TUNEL reaction mixture (TdT-mediated dUTP-X nick end labeling). And then incubated secondary antibody conjugated a biotin to enable visualization of the fluorescent markers for light microscopy.

### FACS analysis

For analysis of subpopulation in the cancer cell lines, the cultured cells were removed from the culture dish with trypsin containing EDTA and washed with PBS. The cells were then labeled with CD133 (Miltenyi Biotec, Germany), CD117 (BD Pharmingen, USA), CD45 (BD Pharmingen, USA), and CD71 (Chemicon, USA) at 4°C for 30 min. Then 1 × 10^5 ^cells were analyzed in a FACSVantage fluorescence-activated cell sorter (Beton Dickinson, San Jose, CA). For an isotype control, nonspecific mouse IgG (BD Pharmingen, USA) was substituted for the primary antibody. After immunocytochemistry of cell surface epitopes we analyzed positive cells using Fluorescence Microscope (Leica Microsystems, PA).

### Immunocytochemistry analysis

For analysis of protein expression of neuronal and glial markers in radio-resistant cancer cells, radio-resistant glioblastoma cells were fixed with 4% paraformaldehyde fixative solution for 30 min at room temperature. After washing with PBS, cells were incubated with primary antibodies against anti-MBP (1:3000, Chemicon, USA), anti-GFAP (1:1500, DAKO Cytomation, Denmark), anti-Nestin (1:200, Sigma, USA), anti-MAP2ab (1:200, Sigma, USA), anti-CD133 (1:10), anti-CD117 (1:10), and anti-CD45 (1:10) overnight at 4°C. After extensive washing with PBS, the cells were incubated for 30 min with FITC, Texas-Red, or TRITC conjugated secondary antibodies (1:250, Molecular Probe, USA). Cell nuclei were labeled with 4–6'diamidino-2-phenylindoline (DAPI; Vector laboratories, UK) and Topro-3 (Molecular Probe, USA). We analyzed using Fluorescence Microscope (Leica Microsystems, PA) and Confocal Microscopy (Leica Microsystems, PA) using a Leica TCS sp2 laser scanning microscope equipped with 3 lasers. Immunocytochemical experiments were repeated at least three times.

### RT-PCR analysis of radio-resistant glioblastoma cells

After gamma-irradiation, cells were harvested with passages, and total cellular RNA was extracted with Trizol (Invitrogen, USA) reverse transcribed into first strand cDNA using oligo-dT primer (Promega, USA) an amplified by 35 cycles (94°C, 1 min; 55°C, 1 min; 72°C, 1 min) of PCR using 20 pM of specific primers. PCR amplification was performed using the primer sets. All primer sequences were determined using established human GeneBank sequences for genes indicative of neural lineages or control genes. Expression of the following molecules was detected by RT-PCR: Nestin (5'-ACCAAGAGACATTCAGACTCC; 3'-CCTCATCCTTATTTTCCACTCC; 302 bp), Sox-2 (5'-TACCTCTTCCTCCCACTCCA; 3'-ACTCTCCTCTTTTGCACCCC; 269 bp), Notch-1 (5'-CAGGCATACCGAGGACTATG; 3'-CAGGCGTGTTGTTCTCACAG; 428 bp), FGFR-1 (5'-GGAGGATCGAGCTCACTCGTGG; 3'-CGGAGAAGTAGGTGGTGTCAC; 428 bp), EGFR (5'-CTTCTTGCAGCGATACAGCTC; 3'-ATGCTCCAATAAATTCACTGC; 441 bp), GFAP (5'-GCTCGATCAACTCACCGCCAAC A; 3'-GGGCAGCAGCGTCTGTCAGGTC; 430 bp), MAP2ab (5'-CAGCAAAGGGATACTTT CAC; 3'-ATGCTTTTTGTTGCTTCTTC; 496 bp), VEGF (5'-ACATCTTCCAGGAGTACCCT GATGAG; 3'-GCATTCACATTTGTTGTGCTGT; 204 bp), and GAPDH(5'-ATCAGCACAGTCCATGCCATCACT; 3'-TGAGGTCCACCACCCTGTTGCTGTA; 460 bp).

### Immunoblot analysis

We performed sonication of cells in 500 μl of lysis buffer (20 mM Tris-HCl (pH 7.5), 150 mM NaCl, 1 mM EDTA, 1% Triton X-100, 2.5 mM sodium pyrophosphate, 1 mM EGTA, 1 mM glycerophosphate, 1 mM Na_3_VO_4_, 1 mM PMSF). Lysates were clarified by centrifugation at 15,000 × g for 10 min and the total protein content was determined. Equal amounts (30 μg) of protein extracts in lysis buffer were subjected to 10% SDS-PAGE analysis and transferred to the nitrocellulose membrane. Anti-PARP, anti-cleaved caspase-3, anti Akt, anti phosphorylated Akt, anti-Erk1/2 and anti phosphorylated-Erk1/2 antibodies were purchased from Cell Signaling Technology Inc. (USA). Anti Bcl 2, and anti Bax antibodies were purchased form Santa Cruz (USA). Anti actin antibody was from Sigma Chemical Co. (USA). Enhanced chemo-luminescence (ECL, Amersham-Pharmacia, UK) system was used for detection. Relative band intensities were determined by quantitation of each band with Quantity-one 1-D analysis software (Bio-Rad, USA).

### In vitro differentiation to neural cells of radioresistant glioblastoma cells

To assess for multipotency, radio-resistant GBM2, A172 and U87MG cells were plated at a density of 2.5 × 10^4 ^cells/cm^2 ^onto fibronection-coated glass coverslips (12 mm diameter) in Neurobasal medium (NB, Invitrogen, USA) containing B27 supplement (Invitrogen, USA), 2% FBS, and 10 ng/ml Retinoic acid (RA, Sigma, USA) for 7 to 10 days according to various time periods (culture passage) of post radiation. Retinoic acid was treated every 2 days.

### Evaluation of tumorigenicity by stereotaxic injection

Tumorigenicity was determined by stereotaxic injection of radio-resistant subpopulation of human glioblastoma multiforme-derived A172 and GBM2 cells. Athymic SCID mice were anesthetized with i.p. ketamine and each cells (5 × 10^5^) were labeled with CFDA-SE cell tracer kit (Chemicon, USA) before transplantation. Cells in 10 ul of HBSS were delivered into the right striatum (0.1 μl/min) by stereotaxic injection through a glass electrode connected to a Hamilton syringe. The following coordinates were used: anterior-posterior = 0; median-lateral = 2.4 mm dorsal-ventral = 2.6 mm. The mice were sacrificed at 4 weeks post-transplantation. The mouse brains were immediately fixed with 4% paraformaldehyde and embedded in Tissue-Tek OCT (optimal culture temperature) compound, and then frozen at -20°C. 10 μm coronal sections were cut through the brain using a freezing microtome (CM3050; Leika Microsystems, PA) and mounted on poly-D-Lysine-coated slides. Hematoxylin and eosin staining and immunohistochemistry were performed on 10 um-thick cryostat sections. Sections were processed as by Vescovi et al. [33].

### Immunohistochemistry on tumor sections

Frozen sections were stained with hematoxylin and eosin (H&E). For visualization of transplanted cells, frozen sections were air-dried, fixed in 4% paraformaldehyde, and mounted in mounting medium containing DAPI. For determine the coexpression of tumor lesions and transplanted human cells by fluorescent staining after immunohistochemistry, frozen sections were stained with anti-mouse von Willebrand factor (1:1000, Chemicon, USA) and anti-human nuclei (1:200, Chemicon, USA) for overnight at 4°C. After washing with PBS, the tumor sections were incubated for 30 min with FITC and TRITC conjugated secondary antibodies (1:250, Molecular Probe, USA). Cell nuclei were labeled with TOPRO-3 (Molecular Probe, USA). Then we analyzed using Fluorescence Microscope (Leica Microsystems, PA) and Confocal Microscopy (Leica Microsystems, PA) using a Leica TCS sp2 laser scanning microscope equipped with 3 lasers.

### Statistical analysis

The statistical significance of difference between groups was calculated by applying the Student's two-tailed *t*-test.

## Authors' contributions

MKK carried out data acquisition, data analysis and interpretation and helped to design the study and draft the manuscript. BIH and MHK carried out part of the experiments on radiation exposure. CHK helped to design the stereotaxic injection of athymic SCID mice. SHC participated in the design and interpretation of the data. SKK conceived of the study, and participated in its design and coordination and drafted the manuscript. All authors read and approved the final manuscript.
